# Perceived benefits and barriers to exercise for recently treated patients with multiple myeloma: a qualitative study

**DOI:** 10.1186/1471-2407-13-319

**Published:** 2013-07-01

**Authors:** Melinda J Craike, Kaye Hose, Kerry S Courneya, Simon J Harrison, Patricia M Livingston

**Affiliations:** 1 Faculty of Health, Deakin University, 221 Burwood Hwy, Burwood, VIC 3125, Australia; 2 Leukaemia Foundation Australia, Ground Floor, 205 Bell St, Preston, VIC 3072, Australia; 3 Behavioral Medicine Laboratory, Faculty of Physical Education and Recreation, E-488 Van Vliet Center, University of Alberta, Edmonton, AB T6G 2H9, Canada; 4 Cancer Medicine, Peter MacCallum Cancer Centre, Locked Bag 1, A’beckett St, Melbourne, VIC 8006, Australia; 5 Sir Peter MacCallum Department of Oncology, University of Melbourne, Parkville, VIC 3010, Australia

**Keywords:** Cancer, Oncology, Multiple Myeloma, Physical Activity, Exercise

## Abstract

**Background:**

Understanding the physical activity experiences of patients with multiple myeloma (MM) is essential to inform the development of evidence-based interventions and to quantify the benefits of physical activity. The aim of this study was to gain an in-depth understanding of the physical activity experiences and perceived benefits and barriers to physical activity for patients with MM.

**Methods:**

This was a qualitative study that used a grounded theory approach. Semi-structured interviews were conducted in Victoria, Australia by telephone from December 2011-February 2012 with patients who had been treated for MM within the preceding 2–12 months. Interviews were transcribed and analysed using the constant comparison coding method to reduce the data to themes. Gender differences and differences between treatment groups were explored.

**Results:**

Twenty-four interviews were completed. The sample comprised 13 females (54%), with a mean age of 62 years (SD = 8.8). Sixteen (67%) participants had received an autologous stem cell transplant (ASCT). All participants currently engaged in a range of light to moderate intensity physical activity; walking and gardening were the most common activities. Recovery from the symptoms of MM and side effects of therapy, psychological benefits, social factors and enjoyment were important benefits of physical activity. Barriers to physical activity predominately related to the symptoms of MM and side effects of therapy, including pain, fatigue, and fear of infection. Low self- motivation was also a barrier. Women participated in a more diverse range of physical activities than men and there were gender differences in preferred type of physical activity. Women were more likely to report psychological and social benefits; whereas men reported physical activity as a way to keep busy and self-motivation was a barrier. Patients treated with an ASCT more often reported affective benefits of participation in physical activity and fatigue as a barrier. Patients treated with other therapies (e.g., chemotherapy, radiotherapy) were more likely to report pain as a barrier.

**Conclusions:**

Patients with MM experience debilitating effects of their condition and therapy, which influences their level and intensity of physical activity participation. Physical activity programs should be individualised; take into consideration gender differences and the impact of different types of therapy on physical activity; and focus on meeting the psychological, coping and recovery needs of patients.

## Background

Multiple myeloma (MM) is an incurable malignancy of plasma cells. In 2010 in Australia, it was estimated that 1,400 people were diagnosed with MM, representing 1.2% of all cancer diagnoses. MM is more common in men than women; the average age at diagnosis is 70 years [1]. Although there is currently no cure for MM, modern therapy can control the disease for prolonged periods and the 5-year survival rates for MM have increased from 26% to 42% from 1985–1989 to 2005–2009 in Victoria, Australia [2].

The preferred therapy for patients with MM depends on their age, functional status and comorbidities. In Australia, guidelines recommend that autologous stem cell transplantation (ASCT) should be the standard of care in patients up to 65–70 years following induction therapy. This therapy comprises an induction regimen incorporating novel agents (thalidomide, bortezomibor or lenalidomide) designed to preserve the capacity to harvest haematopoietic stem cells. Patients older than 65 years with poor performance status, or younger patients with comorbidities are not eligible for ASCT due to increasing toxicity, regimens usually combine melphalan and steroids with novel agents. Supportive therapy may include the use of bisphosphonates and erythropoietin as per updated guidelines [3].

The treatment regimens for MM are complex and demanding [3]. The impact of the underlying disease and the side effects of treatment include chronic pain, fatigue, nausea and vomiting, recurrent infections and anaemia [4,5]. Patients also frequently suffer from osteoporosis and osteolytic bone lesions, putting them at increased risk of pathological fracture [6,7]. These outcomes reduce the quality of life of patients and are often associated with increased incidence of depression, anxiety and distress [4,5,8].

Physical activity has been shown, through randomised controlled trials, to improve physical and psychological outcomes among patients with solid tumours [9-11]. This has led to interest in how participation in physical activity may be facilitated for cancer survivors [12-14]. Physical activity behaviors, and the factors that influence these behaviors, vary by cancer diagnosis [15-17], patient demographics [6,7,15], and stage in the cancer journey [15,16]. Thus it is important to examine the barriers to physical activity and benefits of participation in physical activity for specific cancer groups, such as MM, and at a defined stage in the illness trajectory.

Examination of the specific benefits of physical activity for people with MM is a relatively new area of research, but one that is gaining increasing attention as the prevalence of MM increases and lifestyle behaviors, such as physical activity, are recognised as important factors in overall patient outcomes [18-20]. Research to date, albeit limited, has shown that physical activity is safe and feasible before, during and following treatment for MM; can alleviate some of the side effects of treatment, including fatigue; and can enhance the quality of life of patients [18,21,22]. Despite these promising findings, the pathophysiology of MM and associated therapies may make physical activity uptake and adherence a challenge for this group. Participation in physical activity is lower for people with MM than other cancer types [18,23]. In addition, Coleman et al. reported a high exercise attrition rate of 42% in MM patients who participated in a randomised trial [22].

One way of increasing our understanding of physical activity in the lives of people with MM is to examine participation experiences and the perceived benefits of and barriers to participation. This information is essential to inform the development of evidence-based interventions to encourage physical activity uptake and adherence and to quantify the benefits of physical activity for this group. The aim of this study was to gain new insights in to the physical activity experiences, perceived benefits, and barriers to participation for patients who were treated for MM within the preceding 2–12 months. Due to limited research in this area and the exploratory nature of this study, a qualitative approach that examined physical activity experiences within the context of the patient’s broader life and from the patient’s perspective, was selected.

## Methods

This study was approved by the Human Research Ethics Committee at Deakin University.

### Research participants

Male and female patients who completed treatment for MM were interviewed for this study. Inclusion criteria were people living in Victoria Australia, aged 18 years and over; a diagnosis of symptomatic MM who had completed therapy (chemotherapy, radiotherapy, induction therapy and/or transplant) 2-12 months prior; and with the ability to speak English and complete English-language versions of the patient-completed measures.

### Procedure

A purposive sampling technique was used to select patients who were living in Victoria, Australia. The patient database maintained by the Leukaemia Foundation of Australia was used to identify potential participants. The database was screened for patient names, cancer diagnosis, age and address details as well as approximate date(s) of treatment for MM. Potential participants were sent a cover letter and Participant Information and Consent Form, which provided an overview of the study, eligibility criteria, and an explanation of what participation in the study would involve. If patients deemed themselves eligible and wished to participate, they were asked to complete the consent form and return it. Once received, the interviewer rang the patient to confirm that they met the eligibility criteria and an interview time was arranged.

A self-administered questionnaire was mailed to participants prior to the telephone interview. Participants were asked to complete the questionnaire before completing the interview and could use it as a reference point during the interview. On completion of the interview, participants were asked to return the questionnaire using a reply paid envelope.

Telephone interviews were conducted from December 2011-February 2012. The interviews were conducted by a nurse counsellor with knowledge of MM and extensive experience in conducting interviews with cancer patients. Interviews were conducted by telephone and were recorded (with the permission of participants). Interviews continued until saturation was reached. A summary of the research findings was sent to the participants once the study was completed.

### Measures

The questionnaire completed prior to the interview measured patient and clinical characteristics, including date of birth, highest level of education, postcode, living arrangements, treatment type and length of time since treatment. Current and pre diagnosis physical activity was measured using an adapted version of the Leisure Time Exercise Questionnaire developed by Godin et al. [24,25]. Participants recorded their average weekly physical activity prior to diagnosis (pre diagnosis physical activity) and their average weekly physical activity in the past month (current physical activity). The Leisure Time Exercise Questionnaire assesses average frequency and duration of light (e.g., easy walking), moderate (e.g., brisk walking) and strenuous (e.g., running) physical activity. It has been used in studies of cancer survivors [26,27] and patients with MM [18].

A grounded theory approach was taken in this study [28]. Interviews were semi-structured and follow up questions and probes facilitated a deeper understanding of the participants’ perceptions and experiences of physical activity. The interview prompts focused on participation in physical activity before, during and after treatment and any perceived barriers and benefits of participation. The interview was guided by a series of pre-determined prompts, with flexibility in the order in which they were covered to allow the interview to flow. Prompts included: “Can you describe your participation in exercise before during and after treatment?”; “What things stop or limit your participation in exercise?”; and “What things motivate you to exercise?”

One interviewer conducted all of the interviews. Author one briefed the interviewer about the aims and purpose of the interviews and listened to and gave feedback on interviewing style. Regular meetings were held between author one and the interviewer to discuss important themes, the point at which saturation was reached and any logistical issues.

### Data analysis

Descriptive statistics were used to analyse the questionnaire data, including the demographic and clinical characteristics and participation in physical activity.

In terms of qualitative data, the interviews were transcribed verbatim and the accuracy of the transcripts was verified, with 80% checked by the researchers against the interview recordings. Data from the interviews were analysed using the nVivo software package. Pseudonyms were assigned to participants so that they could not be identified.

The analysis process was inductive and coding was used to reduce the data into meaningful themes [29]. The coding procedures applied the “constant comparison” method [30]. The constant comparison method utilises three stages of coding. For the initial stage, a relevant code was applied to ideas in the transcripts to develop categories which captured the meaning of the idea [31]. Under the supervision of authors one and five, a research assistant coded the data. As a way of validating the codes, three interviews were independently coded by author one to check the interpretations of the coder and validate the themes. There was agreement between both researchers as to the dominant themes and their interpretation of the meaning from the ideas represented in the interviews.

The second stage of coding involved reducing codes through grouping similar codes into broader, more encompassing themes and comparing them to one another and cross checking back to the original interview text. In the final stage, categories were delimited to gain parsimony and focus on the aims of the study [31]. At this stage, comparisons were made based on gender and type of therapy (ASCT or other therapies, including chemotherapy, radiotherapy). Examination of different therapy groups was important as treatment with or without ASCT may influence the functional status of the patient, which may have an impact on their physical activity. During the coding processes, the authors and interviewer met to discuss the themes that were emerging from the interviews.

In the Results section, the gender, age and main treatment type of participants are included in parentheses following direct quotes. Only the main treatment type has been included here; participants may also have been treated with a range of induction and supportive therapies including thalidomide and steroids (e.g., prednisolone and zometa).

## Results

### Sample and clinical characteristics

Thirty-two patients responded to the initial mail out, of which eight did not complete the interview due to ineligibility (i.e. had not received therapy for MM in the past 2–12 months; n = 5), too unwell or emotionally distressed (n = 2), or lack of interest in completing interview (n = 1). In total, 24 interviews were completed, 13 were female (54%). The age of the sample ranged from 48–78 years, with a mean age of 62 years (SD = 8.8; see Table 1).

**Table 1:** Sample and clinical characteristics

	***n *(%)**
	**(*N*** **= 24)**
**Gender**	
Male	11 (46)
Female	13 (54)
**Age**	
Mean (SD)	62 (8.8)
**Living Arrangements**	
Partner/spouse	16 (67)
Partner/spouse and children	6 (25)
Alone	2 (8)
**Highest Level of Education**	
University degree or higher	10 (42)
Certificate or diploma	8 (33)
Secondary school	5 (21)
Primary school	1 (4)
**Region**	
Metropolitan area	11 (46)
Regional/rural area	13 (54)
**Treatment**	
Autologous Stem Cell Transplant	16 (67)
Chemotherapy	6 (25)
Radiotherapy	5 (21)
Other (e.g., steroids, Thalidomide/Revlimid)	17 (71)
**Time Since Treatment Completion**	
2-4 months ago	4 (17)
5-7 months ago	9 (37.5)
8-10 months	2 (8)
11-12 months	4 (17)
Over 12 months	1 (4)
Ongoing (e.g., thalidomide)	4 (17)

The majority of participants lived with either a partner/spouse 16 (67%); or a partner/spouse and children (own or partners) (*n* = 6; 25%). In terms of highest level of education, 10 (42%) had a University degree or higher and 8 (33%) had a certificate or diploma. There were more participants from regional/rural areas (n = 13, 54%) than metropolitan areas (n = 11, 46%).

Two-thirds of participants had been treated with a stem cell transplant (*n* = 16; 67%) and most participants had completed treatment 5–7 months ago (*n* = 9; 37.5%), followed by 2–4 months ago (*n* = 4; 17%) or 11-12 months (*n* = 4; 17%).

### Current participation in physical activity and change from Pre-diagnosis

#### ***Current type and intensity of physical activity***

None of the participants had participated in vigorous intensity physical activity on an average week in the past month; 56.2% participated in some moderate intensity physical activity (*M* = 84 minutes per week, *SD* = 104.9); and 69.6% participated in some light intensity physical activity (*M* = 85 minutes per week, *SD* = 85.9). Overall 26% of participants were meeting the recommended guidelines of 150 minutes of moderate-vigorous intensity physical activity per week.

Walking, followed by gardening were the most common physical activities. A range of other activities were also discussed, including bike riding, yoga, swimming, stretching, tennis, pilates, tai chi, table tennis and strength training. Most of these activities were of light to moderate intensity. Participants also spoke about trying to increase their level of physical activity after their therapy. For some, this meant increasing the length of time they walked each day. Participants also spoke about their level of physical activity varying depending on how they were feeling, which was a function of their health and motivation as well as external factors like the weather.

*…Yeah, well I try to walk every day. I’ve never been really a sporty person but I’ve always enjoyed walking prior to my myeloma and all of that. I enjoy gardening a lot. So as I say, I try to walk, I won’t say every day but probably five out of seven days a week and I’ll definitely go off for about 30 to 60 minutes, depends on the day and the weather and how I’m feeling, what sort of energy I’m at, that sort of level*. (‘Francis^a^’, Female, 54 years, treated with a stem cell transplant)

#### ***Change from physical activity prior to diagnosis***

Most participants in the interviews reported that the intensity and/or frequency of physical activity had reduced since their diagnosis. This was consistent with the questionnaire data which showed that participation in moderate and vigorous physical activity had reduced. Prior to diagnosis, 21.7% participated in vigorous physical activity and the mean number of minutes per week was 32.6 minutes (compared to 0 minutes now); 60.9% had participated in moderate physical activity and the mean number of minutes per week was 103.6 minutes (compared to 56.2% and 84 minutes per week now). The percent of participants who were currently participating in light intensity physical activity was similar to prior to diagnosis (69.6% compared to 65.2%) and average minutes were similar at 71.1 minutes (prior to diagnosis) and 85 minutes per week now. Note that the pre-diagnosis moderate and light intensity physical activity minutes per week were not included for one participant whose response was invalid.

Some participants were not able to do any sort of physical activity; while others continued with lighter intensity or less frequent physical activity compared to before their diagnosis, as illustrated in the following quote.

*Yeah, look, I wouldn't be doing as intense exercise as I was previously. I physically probably can't do it to the same level that I had. So in terms of quantity it's probably dropped off slightly but there hasn't been a large difference there. It's probably more just the intensity at which I do it*. (‘Michael’, Male, 48 years, treated with a stem cell transplant)

There were some participants who were back to or close to their pre diagnosis level of physical activity and two actually participated in more physical activity now.

Both men and women participated in walking, however there were gender differences in other types of physical activity. Women participated in a wider range of activities than men and were more likely to report participating in aquatics, gym work, pilates, yoga and Tai Chi. Men were more likely to report participation in golf, gardening, bike riding and bowls.

### Perceived benefits of physical activity

Almost all respondents reported that physical activity was beneficial to them. These benefits were reported as predominantly related to their recovery from treatment and coping with symptoms of MM as well as psychological benefits. Other benefits included physical improvements such as appearance, weight loss, enjoyment and social interaction.

#### ***Recovery from treatment/disease***

*Recovery* was defined by participants in terms of physical and psychological recovery and also getting back in to a normal routine that they enjoyed prior to their diagnosis. Physical recovery was focused on the prevention of deterioration in physical health and function. There was a sense that physical activity facilitated participants to regain a routine and normality after what, for many, was a traumatic diagnosis and treatment regimen.

*I think it gives you a more positive outlook if anything. You can get back to normality and you can do things you know*…..(‘Sophie’, Female, 57 years, treated with a stem cell transplant)

Not all participants, however, saw the benefits of exercise in their recovery because the symptoms that they experienced were so extreme and constant that nothing seemed to relieve them:

*Well I'd like to think that it was helping. But it doesn't seem to be - I just don’t seem to be able to do anything that is helping it [pain in lower body]. …… It's consistently there all the time, lesser or more, depending on the time of day. The more I seem to walk, the worse it gets*. (‘Frank’, Male, 64, treated with a stem cell transplant)

#### ***Psychological health***

Many participants spoke about the psychological benefits of physical activity. This was more prevalent than the discussion of physical health benefits. There were several dimensions to the perceived psychological health benefits, these included (1) cognitive improvements of being more alert, keeping the mind healthy and fresh, and improvements in concentration; and (2) affective changes including feeling good, a sense of accomplishment, helping emotionally, helping to cope with MM.

In terms of cognitive improvement:

*Well I suppose it just keeps you going. Certainly my job is very sedentary and I know that if I don't start the day with some exercise it's harder to concentrate and things like that*…(‘Anne’, Female, 58 years, treated with a stem cell transplant)

Affective changes that were bought on through physical activity, including feeling better and keeping the mind from worrying are demonstrated through this quote:

*Oh. Put it this way, I was quite depressed when I was in the house after the stem cells transplant and, of course, after the two VAD chemo. I find myself if I go out and did a bit of walking, I feel a little bit better, so I go from there*. (‘Jane’, Female, 60 years, treated with a stem cell transplant)

Although the psychological benefits were noted among both men and women, there were some differences. Men reported being motivated to ‘do something’ and keep busy, whereas women more often reported the psychological benefits of physical activity and feeling better. Affective changes, particularly ‘feeling good’ were more often reported by those who had an ASCT compared to other those who had other types of therapy.

#### ***Enjoyment***

Most of the participants said they enjoyed being physically active; this was often intertwined with the psychological benefits of participation and ‘feeling better’ and also the sense of accomplishment that comes with being physically active:

*Oh I enjoy it actually - particularly a long walk - as I said before 300 odd kilometres - when you finish a walk like that you feel very - as if you've accomplished something really good*. (‘Andrew’, Male, 78 years, treated with Thalidomide)

#### ***Social factors***

Social factors were another motivation for physical activity. Although some participants enjoyed being physically active and having time to themselves, others saw it as an opportunity to be involved with family and friends. Physical activity was also seen as a way of connecting with other people, avoiding isolation, getting back in to life after MM and making new friendships. This could be with people with MM or other friends. Both men and women enjoyed the social aspects of participation; however this was discussed more by women than men.

*I'm always better to have done exercise in a group, basketball, netball, all of that. The oncology rehab, going there twice a week and the girls right now we'll go for a walk, now we'll do this, now we'll do that. I need someone to push me a little*. (‘Tina’, Female, 65 years, treated with a stem cell transplant)

### Perceived barriers to physical activity

#### ***MM symptoms and side effects of treatment***

The most prevalent barriers to physical activity were symptoms of MM and side effects of treatment. Barriers either limited physical activity or stopped it completely. These included fatigue (low energy levels, little stamina and tiredness); pain (particularly bone pain); concerned about bone fractures/bones; low immune system and subsequent fear of infections in public exercise locations, particularly gyms; taking medications in a timely manner; self-conscious about appearance after treatment (weight gain, loss of hair); anaemia; back pain; and foot weakness.

Fatigue, both physical and mental, was the most prominent barrier. Participants also experienced effects on concentration and keeping their mind focused on tasks, and sometimes felt ‘unsure about what you are doing’. One participant described this as having a ‘tired head’. Participants reported that fatigue was felt over their entire body and they experienced extreme tiredness.

*… I get tired. I’m ready for bed at 8:30, nine o’clock every night, you know…as soon as the sun goes down I’m ready for bed and I’ll go and jump into bed… So I don’t know whether it’s the drugs or whether it’s the myeloma. The physicians told me it’s probably mainly the drugs that has done it. Hopefully the myeloma is under control at the moment*. (‘Charles’, Male, 51 years, treated with chemotherapy)

Pain was also a barrier that limited or prohibited physical activity. This pain was mostly related to bone pain in various parts of the body, including the back, neck, elbows and hips. As pain levels were more debilitating on some days than others, it had the effect of either limiting or completely preventing any sort of physical activity. The intensity and ongoing nature of the pain was wearing on participants and made it difficult to continue on with their day to day life.

*… I’ve got pains in the back and look, I’m all right at the moment, touch wood, but you know, pains in the back and hip, one of my elbows and they’re just sort of – I don’t know, it just grinds you down, I suppose, and makes you come to a stop or in my case anyway*. (‘Charles’, Male, 51 years, treated with chemotherapy)

However, pain was not experienced as much by some participants, who felt that it was not a barrier for them:

*I'm lucky. I don’t feel that much pain related to the myeloma, but if I'm really painful, well, I'll stop a day or two and see how it goes. If it doesn't go away, I know something's wrong, so I go to see my doctor*. (‘Jane’, Female, 60 years, treated with a stem cell transplant)

Fatigue was more commonly reported by people who had been treated with an ASCT; however pain as a barrier to physical activity was more often reported by people who had been treated with other types of therapy such as chemotherapy and/or radiotherapy.

#### ***Low self motivation and lack of interest in physical activity***

Low self motivation and interest in physical activity were barriers identified by participants. Low self motivation was identified by participants who may have had an interest in being involved in physical activity, however they experienced a general lack of motivation. This was intertwined with finding it difficult to ‘get going’, particularly in the morning. Males reported having low self motivation more often than females. Low interest in physical activity was more related to lack of interest in physical activity itself and therefore not participating.

*…. two things that stop me probably doing a lot of exercise. One is I probably wouldn't be interested in it but the other one - I don’t have any problem with my heart, I'm sure I could do a bit of jogging or running, but it's no interest to me*. (‘Dean’, Male, 65 years, treated with a stem cell transplant)

## Discussion

The purpose of this study was to explore the physical activity experiences of people with MM and perceived benefits and barriers to participation. Patients overwhelmingly reported that physical activity was beneficial; the most prominent benefits were in symptom control and recovery from the side affects of MM therapy and the psychological benefits of participation. The main barriers to physical activity related to the symptoms of MM and side effects of therapy and low self-motivation. There were some gender differences in type of physical activity that participants engaged in and benefits and barriers; and there were also some differences in benefits and barriers according to type of therapy.

Participation in physical activity decreased since prior to diagnosis, confirming the findings of previous research with MM patients [18,23] and other cancer survivors [32,33]. Physical activity was of light to moderate intensity, and walking was the most popular type of activity, followed by gardening. These findings are similar to population-based studies, which show that walking is the most popular physical activity among older adults [34]. Walking was the most popular physical activity for both men and women, however there were some differences between men and women in physical activity participation. For example, women participated in aquatics, gym work and pilates, whereas men participated in golf, gardening and bike riding. These are consistent with gender differences in physical activity participation in the general population of adults and older adults [34,35].

One-quarter of participants were meeting the recommended guidelines of 150 minutes of moderate-vigorous intensity physical activity per week; this was similar to previous studies of people with multiple myeloma [25,36]. This compares with 30-45% of other cancer survivor groups who met the guidelines for sufficient levels of physical activity [6,18,30]. Our findings reinforce that MM may be more debilitating than some other types of cancer, which represents additional challenges to performing regular physical activity for people with MM.

None of the participants from this study engaged in vigorous physical activity. We found that participating in light to moderate intensity physical activity is likely to be the most feasible for patients with MM who experience a range of physical limitations that effect mobility, and who are also at increased risk of bone fractures and infections [6,7]. These findings suggest that patients with MM may find it difficult to meet the American College of Sports Medicine guidelines for cancer survivors, which advise that cancer survivors avoid inactivity and follow the age-appropriate guidelines for aerobic activity; the accumulation of 150 minutes per week of moderate to vigorous intensity physical activity. However, in recognition of the specific needs of some cancer groups, the panel acknowledged that there should be some cancer site-specific alterations for patients and caution was advised for those at increased risk of fracture and infection [37].

In this study, the social context of the physical activity was important for people with MM and social interactions were important to the overall physical activity experience; this was particularly so for women. This finding supports the findings of previous research which demonstrates that having an exercise role model or partner is positively associated with physical activity participation for patients with MM [38], as well as other cancer groups, including prostate cancer survivors [39] and breast cancer survivors [40].

One of the main reported benefits of physical activity was helping to overcome the impact of MM treatment and symptoms. This might be a mechanism through which physical activity contributes to quality of life and psychological health, as previous research has demonstrated the association between symptom distress, quality of life and depression [41].

The psychological benefits of physical activity, including cognitive, affective and coping with cancer were frequently reported by interview participants in our study. These psychological benefits are particularly important for people with MM, as depression and low quality of life are frequently reported [4,8]. Although no randomised controlled trials have been conducted with patients with MM, two recent meta analyses of studies of cancer survivors (primarily breast cancer) concluded that physical activity had a positive effect on psychological health [42,43]. However, other studies have shown no association between physical activity and depression and anxiety for breast cancer survivors [10,44] or colorectal cancer survivors [45].

There is evidence that the intensity of physical activity plays an important role in outcomes [46]. The effect of level of intensity on quality of life and psychological health outcomes is complex and there is debate about the optimal intensity, particularly for psychological health [47]. A cross-sectional study of patients with MM by Jones et al. showed that during off treatment periods, minutes of participation in moderate plus vigorous intensity physical activity, was associated with overall quality of life and all components of quality of life except physical wellbeing, as well as reductions in fatigue and depression [18]. Further examination of the effect of physical activity at various intensity levels on psychological health and quality of life outcomes for patients with MM is warranted.

Symptoms of MM and side effects of treatment, particularly fatigue and pain, were the predominant barriers to physical activity. Evidence suggests that symptoms of fatigue, sleep disturbances, pain and loss of appetite were significantly worse for MM patients than those with lymphoma [48]. Fatigue and pain have been identified as barriers to physical activity in other studies of cancer survivors [27,49] and people with MM [23]. Research has shown that higher levels of fatigue are associated with lower levels of physical activity for patients with MM [18]. However, a small randomised controlled trial by Coleman et al. demonstrated that physical activity reduced fatigue for patients with MM [21]. We found that the extent to which pain and fatigue were barriers to participation differed by treatment type, with pain experienced more by people who had been treated with therapies including chemotherapy and/or radiotherapy and fatigue experienced more by people who had a ASCT. These associations and their impact on physical activity experiences requires further investigation.

Lack of self-motivation was also a barrier in our study, particularly for men and for those who were treated with chemotherapy and/or radiotherapy. Lack of self-motivation has also been identified in other studies of cancer survivors [49].

The strengths of this study were the inclusion of people with MM who were recently treated, which facilitated recall of physical activity prior to diagnosis and the experience of treatment. The selection of participants from a population-based database increased the possibility of gaining perspectives from people from a range of backgrounds and localities.

Limitations of the study also need to be considered when interpreting the findings. This study was cross sectional and comprised a small sample size, involving younger patients (mean age = 62 years) than the population of MM patients (mean age at diagnosis of 70 years [1]) and findings can therefore not be generalized to the population. Participants were at least somewhat physically active; with voluntary participation, this self-selection bias is difficult to avoid. Participants had difficulty recalling their treatment regimen and we are not able to verify the accuracy of patient treatment status. The measure of pre-treatment level of physical activity was retrospective, which increases the possibility of recall error [50]. However, the main focus of this study was on the participants’ description and lived experience of physical activity. Given these limitations, the findings of our study should be further examined through a population-based quantitative study examining the determinants of physical activity and potential outcomes such as improved quality of life (particularly levels of fatigue and pain), anxiety and depression.

## Conclusions

Patients with MM predominantly participate in light to moderate intensity physical activity; this may be at least partly attributed to the side effects of their condition and treatment. Physical activity programs should focus on meeting the psychological and recovery needs of patients, while being conscious of the limitations that are faced by people with MM. An individualised program design that considers gender and treatment related differences is warranted. The involvement of specialists who understand MM is important so that side effects and cancer symptoms are taken in to account in the design of physical activity programs.

### Endnotes

^a^Participant pseudonyms have been used.

## Competing interests

The authors declare that they have no competing interests.

## Authors’ contributions

MJC conceived of the study, participated in its design and coordination, contributed to the data analysis and interpretation and drafted the manuscript. KH contributed to the study design, development of interview questions, participant recruitment and assisted in the drafting of the manuscript, PML contributed to the study design, data analysis and interpretation and drafting of the manuscript; KSC contributed to the study design, drafting of interview prompts, interpretation and drafting of the manuscript; SJH contributed to the study design, drafting of the manuscript and provided expert advice on MM and treatments. All authors read and approved the final manuscript.

## Pre-publication history

The pre-publication history for this paper can be accessed here:

http://www.biomedcentral.com/1471-2407/13/319/prepub

## References

[B1] Australian Institute of Health and Welfare Cancer in Australia: An overview 2010 Canberra, Australia: AIHW

[B2] Thursfield V Farrugia H Cancer in Victoria: Statistics and trends 2010 2011 Melbourne, Australia: Cancer Council Victoria

[B3] Medical Scientific Advisory Group to the Myeloma Foundation of Australia Clinical Practice Guidelines: Multiple Myeloma 2012 Australia: Myeloma Foundation of Australia

[B4] Molassiotis A Wilson B Blair S Howe T Cavet J Unmet supportive care needs, psychological well-being and quality of life in patients living with multiple myeloma and their partners Psychooncology 2011 20 1 88 97 10.1002/pon.1710 20187072

[B5] Sherman A Simonton S Latif U Tricot G Psychosocial adjustment and quality of life among multiple myeloma patients undergoing evaluation for autologous stem cell transplantation Bone Marrow Transplant 2004 33 9 955 962 10.1038/sj.bmt.1704465 15034542

[B6] Seidl S Kaufmann H Drach J New insights into the pathophysiology of multiple myeloma Lancet Oncol 2003 4 557 564 10.1016/S1470-2045(03)01195-1 12965277

[B7] Drake MT Bone disease in multiple myeloma Oncology 2009 23 14, Suppl 5 28 32 20128326

[B8] Sherman AC Simonton S Latif U Plante TG Anaissie EJ Changes in quality-of-life and psychosocial adjustment among multiple myeloma patients treated with high-dose melphalan and autologous stem cell transplantation Biol Blood Marrow Transplant 2009 15 1 12 20 10.1016/j.bbmt.2008.09.023 19135938

[B9] Galvão DA Taaffe DR Spry N Joseph D Newton RU Combined resistance and aerobic exercise program reverses muscle loss in men undergoing androgen suppression therapy for prostate cancer without bone metastases: A randomized controlled trial J Clin Oncol 2010 28 2 340 347 10.1200/JCO.2009.23.2488 19949016

[B10] Courneya KS Segal RJ Mackey JR Gelmon K Reid RD Friedenreich CM Ladha AB Proulx C Vallance JKH Lane K Yasui Y McKenzie DC Effects of aerobic and resistance exercise in breast cancer patients receiving adjuvant chemotherapy: A multicenter randomized controlled trial J Clin Oncol 2007 25 28 4396 4404 10.1200/JCO.2006.08.2024 17785708

[B11] Segal RJ Reid RD Courneya KS Malone SC Parliament MB Scott CG Resistance exercise in men receiving androgen deprivation therapy for prostate cancer J Clin Oncol 2003 21 9 1653 1659 10.1200/JCO.2003.09.534 12721238

[B12] Courneya KS Stevinson C McNeely ML Sellar CM Friedenreich CM Peddle-Mcintyre CJ Chua N Reiman T Effects of supervised exercise on motivational outcomes and longer-term behavior Med Sci Sports Exer 2012 44 3 542 549 10.1249/MSS.0b013e3182301e06 21814149

[B13] Livingston P Salmon J Courneya K Gaskin C Craike M Botti M Broadbent S Kent B Efficacy of a Referral and Physical Activity Program for Survivors of Prostate Cancer [ENGAGE]: Rationale and design for a cluster randomised controlled trial BMC Cancer 2011 11 1 237 10.1186/1471-2407-11-237 21663698 PMC3141765

[B14] Gjerset GM Fossa SD Courneya KS Skovlund E Thorsen L Exercise behavior in cancer survivors and associated factors J Cancer Surviv 2011 5 1 35 43 10.1007/s11764-010-0148-4 20890674 PMC3040309

[B15] Karvinen KH Raedeke TD Arastu H Allison RR Exercise programming and counseling preferences of breast cancer survivors during or after radiation therapy Oncol Nurs Forum 2011 38 5 E326 E334 10.1188/11.ONF.E326-E334 21875828

[B16] Jones LW Guill B Keir ST Carter K Friedman HS Bigner DD Reardon DA Exercise interest and preferences among patients diagnosed with primary brain cancer Support Care Cancer 2007 15 1 47 55 10.1007/s00520-006-0096-8 16819629

[B17] Jones LW Courneya KS Vallance JK Ladha AB Mant MJ Belch AR Reiman T Understanding the determinants of exercise intentions in multiple myeloma cancer survivors: an application of the theory of planned behavior Cancer Nurs 2006 29 3 167 10.1097/00002820-200605000-00001 16783115

[B18] Jones LW Courneya KS Vallance JKH Ladha AB Mant MJ Belch AR Stewart DA Reiman T Association between exercise and quality of life in multiple myeloma cancer survivors Support Care Cancer 2004 12 11 780 788 10.1007/s00520-004-0668-4 15322968

[B19] Paul KL Rehabilitation and exercise considerations in hematologic malignancies Am J Phys Med Rehabil 2011 90 S76 S82 10.1097/PHM.0b013e31820be0d1 21765268

[B20] Rome S Jenkins B Lilleby K Mobility and safety in the multiple myeloma survivor Clin J Oncol Nurs 2011 15 41 52 21816709 10.1188/11.S1.CJON.41-52

[B21] Coleman EA Coon S Hall-Barrow J Richards K Gaylor D Stewart B Feasibility of exercise during treatment for multiple myeloma Cancer Nurs 2003 26 5 410 419 10.1097/00002820-200310000-00012 14710804

[B22] Coleman EA Coon SK Kennedy RL Lockhart KD Stewart CB Anaissie EJ Barlogie B Effects of exercise in combination with epoetin alfa during high-dose chemotherapy and autologous peripheral blood stem cell transplantation for multiple myeloma Oncol Nurs Forum 2008 35 3 E53 E61 10.1188/08.ONF.E53-E61 18467280 PMC2447545

[B23] Craike M Hose K Livingston PM Physical activity participation and barriers for people with multiple myeloma Support Care Cancer 2012 21 1 8 23052913 10.1007/s00520-012-1607-4

[B24] Godin G Jobin J Bouillon J Assessment of leisure time exercise behavior by self-report: A concurrent validity study Can J Public Health 1986 77 5No. 5 359 361 3791117

[B25] Godin G Shephard RJ A simple method to assess exercise behaviour in the community Can J Appl Sport Sci 1985 10 141 146 4053261

[B26] Trinh L Plotnikoff RC Rhodes RE North S Courneya KS Associations between physical activity and quality of life in a population-based sample of kidney cancer survivors Cancer Epidemiol Biomarkers Prev 2011 20 5 859 868 10.1158/1055-9965.EPI-10-1319 21467240

[B27] Courneya KS Friedenreich CM Quinney HA Fields ALA Jones LW Vallance JKH Fairey AS A longitudinal study of exercise barriers in colorectal cancer survivors participating in a randomized controlled trial Ann Behav Med 2005 29 147 153 10.1207/s15324796abm2902_9 15823788

[B28] Glaser B Strauss A The discovery of grounded theory 1967 Chicago: Aldine Publishing Co

[B29] Strauss AL Corbin J Basics of Qualitative Research: Grounded Theory Procedures and Techniques 1990 Newbury Park, CA: Sage Publications

[B30] Glaser B Strauss AL The Discovery of Grounded Theory 1967 Chicago: Aldine Publishing Co

[B31] Miles M Huberman AM Qualitative Data Analysis 1994 Thousand Oaks, CA: Sage Publications

[B32] Hawkes A Lynch B Youlden D Owen N Aitken J Health behaviors of Australian colorectal cancer survivors, compared with noncancer population controls Support Care Cancer 2008 16 10 1097 1104 10.1007/s00520-008-0421-5 18347819

[B33] Littman A Tang M-T Rossing M Longitudinal study of recreational physical activity in breast cancer survivors J Cancer Surviv 2010 4 2 119 127 10.1007/s11764-009-0113-2 20180037

[B34] Australian Bureau of Statistics Participation in Sport and Physical Recreation 2009–10 2010 In. Canberra: Australian Bureau of Statistics

[B35] Dafna M Carmen C Kamalesh V Adrian B How diverse was the leisure time physical activity of older Australians over the past decade? J Sci Med Sport 2012 15 3 213 219 10.1016/j.jsams.2011.10.009 22197582

[B36] Craike M Hose K Livingston P Physical activity participation and barriers for people with multiple myeloma Support Care Cancer 2013 21 4 927 934 10.1007/s00520-012-1607-4 23052913

[B37] Schmitz KH Courneya KS Matthews C Demark-Wahnefried W Galvao DA Pinto BM Irwin ML Wolin KY Segal RJ Lucia A Schneider CM von Gruenigen VE Schwartz AL American College of Sports Medicine roundtable on exercise guidelines for cancer survivors Med Sci Sports Exerc 2010 42 1409 1426 10.1249/MSS.0b013e3181e0c112 20559064

[B38] Coon SK Coleman EA Keep moving: patients with myeloma talk about exercise and fatigue Oncol Nurs Forum 2004 31 6 1127 1135 10.1188/04.ONF.1127-1135 15547635

[B39] Craike M Livingston PM Botti M An exploratory study of the factors that Influence physical activity for prostate cancer survivors Support Care Cancer 2011 19 7 1019 1028 10.1007/s00520-010-0929-3 20623146

[B40] Hsu H-T Dodd MJ Guo S-E Lee KA Hwang S-L Lai Y-H Predictors of exercise frequency in breast cancer survivors in Taiwan J Clin Nurs 2011 20 13–14 1923 1935 21615573 10.1111/j.1365-2702.2010.03690.x

[B41] McMillan S Tofthagen C Morgan M Relationships among pain, sleep disturbances, and depressive symptoms in outpatients from a comprehensive cancer center Oncol Nurs Forum 2008 35 4 603 611 10.1188/08.ONF.603-611 18591165

[B42] Fong DYT Ho JWC Hui BPH Lee AM Macfarlane DJ Leung SSK Cerin E Chan WYY Leung IPF Lam SHS Taylor AJ Cheng K-k Physical activity for cancer survivors: meta-analysis of randomised controlled trials BMJ 2012 344 E70 10.1136/bmj.e70 22294757 PMC3269661

[B43] Brown JC Huedo-Medina TB Pescatello LS Ryan SM Pescatello SM Moker E LaCroix JM Ferrer RA Johnson BT The efficacy of exercise in reducing depressive symptoms among cancer survivors: a meta-analysis PLoS One 2012 7 1 E30955 10.1371/journal.pone.0030955 22303474 PMC3267760

[B44] Cadmus L Alvarez-Reeves M Mierzejewski E Latka R Saucier L Stoddard C Irwin M Effect of exercise on quality of life during and after treatment for breast cancer: results of two randomized controlled trials AACR Meeting Abstracts 2006 2006 3 A169

[B45] Chambers SK Lynch BM Aitken J Baade P Relationship over time between psychological distress and physical activity in colorectal cancer survivors J Clin Oncol 2009 27 10 1600 1606 10.1200/JCO.2008.18.5157 19255326

[B46] Chang Y Etnier JL Exploring the dose–response relationship between resistance exercise intensity and cognitive function J Sport Exerc Psychol 2009 31 5 640 656 20016113 10.1123/jsep.31.5.640

[B47] Asztalos M De Bourdeaudhuij I Cardon G The relationship between physical activity and mental health varies across activity intensity levels and dimensions of mental health among women and men Public Health Nutr 2009 13 8 1207 1214 20018121 10.1017/S1368980009992825

[B48] Anderson KC Giralt S Mendoza T Symptom burden in patients undergoing autologous stem-cell transplantation Bone Marrow Transplant 2007 39 12 759 766 10.1038/sj.bmt.1705664 17438588

[B49] Blaney JM Lowe-Strong A Rankin-Watt J Campbell A Gracey JH Cancer survivors' exercise barriers, facilitators and preferences in the context of fatigue, quality of life and physical activity participation: a questionnaire–survey Psychooncology 2011 22 186 194 23296635 10.1002/pon.2072

[B50] Lynch BM Owen N Newman BM Pakenham K Leggett B Dunn J Aitken J Reliability of a measure of prediagnosis physical activity for cancer survivors Med Sci Sports Exer 2006 38 4 715 719 10.1249/01.mss.0000210196.94952.8b 16679988

